# TCA cycle-derived oncometabolites in cancer and the immune microenvironment

**DOI:** 10.1186/s12929-025-01186-y

**Published:** 2025-09-10

**Authors:** Shukla Sarkar, Chien-I Chang, Jussekia Jean, Meng-Ju Wu

**Affiliations:** Division of Gastroenterology, Department of Medicine, University of Massachusetts Chan Medical School, Worcester, MA USA

**Keywords:** Oncometabolites, 2-hydroxyglutarate, Succinate, Fumarate, Itaconate, α-ketoglutarate, Epigenetic regulation, Tumor immunity, Metabolic reprogramming, TCA cycle

## Abstract

Oncometabolites are aberrant metabolic byproducts that arise from mutations in enzymes of the tricarboxylic acid (TCA) cycle or related metabolic pathways and play central roles in tumor progression and immune evasion. Among these, 2-hydroxyglutarate (2-HG), succinate, and fumarate are the most well-characterized, acting as competitive inhibitors of α-ketoglutarate-dependent dioxygenases to alter DNA and histone methylation, cellular differentiation, and hypoxia signaling. More recently, itaconate, an immunometabolite predominantly produced by activated macrophages, has been recognized for its dual roles in modulating inflammation and tumor immunity. These metabolites influence cancer development through multiple mechanisms, including epigenetic reprogramming, redox imbalance, and post-translational protein modifications. Importantly, their effects are not limited to cancer cells but extend to various components of the tumor microenvironment, such as T cells, macrophages, dendritic cells, and endothelial cells, reshaping immune responses and contributing to immune suppression. In this review, we highlight the emerging insights into the roles of TCA cycle-associated oncometabolites in cancer biology and immune regulation. We discuss how these metabolites impact both tumor-intrinsic processes and intercellular signaling within the tumor microenvironment. Finally, we examine therapeutic strategies targeting oncometabolite pathways, including mutant IDH inhibitors, α-ketoglutarate mimetics, and immunometabolic interventions, with the goal of restoring immune surveillance and improving cancer treatment outcomes.

## Introduction

Cancer is known for its significant shift in metabolism, a key feature that helps tumors grow, survive, and evade the immune system [1]. One key pathway altered in this process is the tricarboxylic acid (TCA) cycle, also known as the Krebs or citric acid cycle. Normally, the TCA cycle functions in mitochondria to oxidize acetyl-CoA, derived from nutrients like glucose, fatty acids, and amino acids, producing reducing equivalents (NADH and FADH₂), which fuel oxidative phosphorylation for ATP generation. In addition to energy production, the TCA cycle supplies critical intermediates for biosynthesis, including nucleotides, amino acids, and lipids.

The TCA cycle is tightly regulated by cellular energy status: high ATP and NADH levels inhibit its activity, while low energy states (ADP, NAD⁺) and calcium stimulate key enzymes such as citrate synthase, isocitrate dehydrogenase (IDH), and α-ketoglutarate dehydrogenase. However, in cancer, mutations in TCA cycle enzymes or alterations in the tumor microenvironment can disrupt this balance, leading to the buildup of abnormal metabolites known as oncometabolites [2, 3].

Oncometabolites Such as 2-hydroxyglutarate (2-HG), fumarate, and succinate promote tumor development by interfering with epigenetic regulation, signaling pathways, and cellular differentiation [4, 5]. In contrast, alpha-ketoglutarate (α-KG), a normal TCA cycle intermediate, exerts tumor-suppressive effects by supporting dioxygenase activity involved in DNA and histone demethylation. Importantly, many oncometabolites exert their pathogenic effects by competitively inhibiting these α-KG-dependent enzymes [6, 7]. Beyond their effects on cancer cells, these metabolites influence the tumor microenvironment (TME) by modulating immune cell function and promoting immune escape [8]. The intersection of metabolism and immunity is a rapidly expanding area of cancer research. Immune cells, such as T cells, macrophages, and natural killer (NK) cells, are metabolically dynamic and respond to cues in their environment. Oncometabolites can suppress immune responses by altering gene expression and inflammatory signaling, leading to immune dysfunction or tolerance within the TME. Itaconate, a metabolite produced primarily by activated macrophages via decarboxylation of cis-aconitate, has recently emerged as an important immunoregulatory molecule. Rather than promoting tumor growth, itaconate modulates oxidative stress and inflammatory gene expression, thereby shaping macrophage activation and influencing immune responses within the TME [9, 10].

In this review, we examine the roles of key TCA-associated metabolites, including α-KG, 2-HG, succinate, fumarate, and itaconate, in cancer development and immune regulation. We explore their cell-autonomous and non-cell-autonomous effects, and highlight emerging therapeutic strategies aimed at reversing their impact to restore immune surveillance and improve cancer treatment outcomes.

## Oncometabolites in cancer

**Fig. 1:**
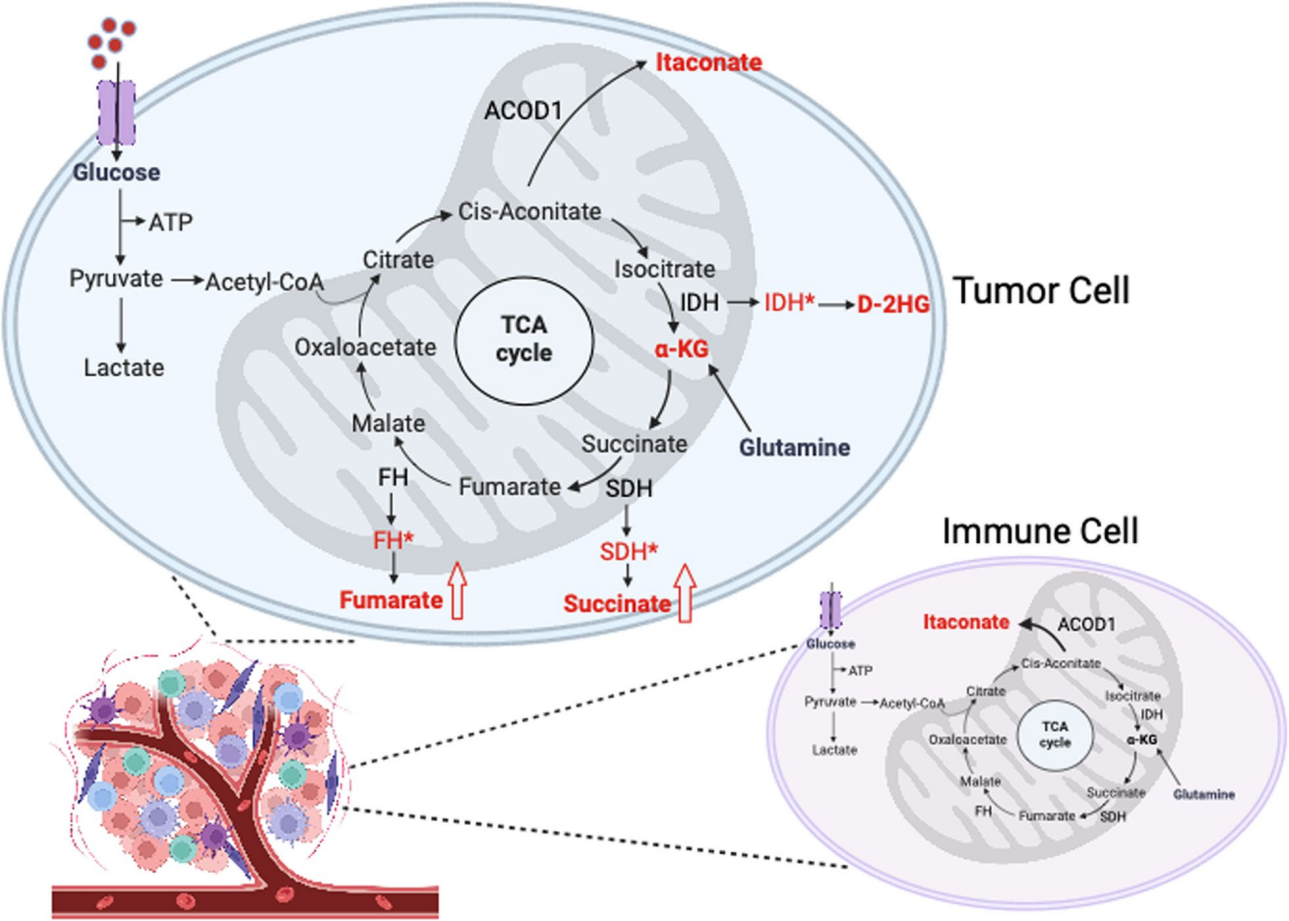
Oncometabolite-driven reprogramming in the TCA cycle. This schematic shows how mutations in TCA cycle enzymes lead to the buildup of oncometabolites that rewire tumor metabolism. Mutant IDH converts α-ketoglutarate (α-KG) into 2-hydroxyglutarate (2-HG), while loss of SDH or FH causes succinate and fumarate accumulation. Separately, immune cells and tumor cells produce itaconate from cis-aconitate by ACOD. These oncometabolites drive cancer progression by altering metabolism, gene regulation, and immune responses

Metabolic reprogramming is a hallmark of cancer and often leads to the abnormal accumulation of metabolites that promote tumor progression and immune Suppression. These molecules, known as oncometabolites, are frequently the result of mutations in metabolic enzymes or altered microenvironmental conditions. This section focuses on key TCA-related oncometabolites: 2-HG, succinate, fumarate, and itaconate. While α-KG itself is not an oncometabolite, it is discussed due to its central role as a counterbalance to the pathogenic effects of these molecules (Fig. 1).

### α-ketoglutarate and 2-hydroxyglutarate

α-KG is a central TCA cycle intermediate generated from isocitrate by isocitrate dehydrogenase (IDH1/2). It functions as a cofactor for a wide range of α-KG–dependent dioxygenases, including TET DNA demethylases and Jumonji-domain histone demethylases. These enzymes regulate gene expression and cell fate decisions.

2-HG, a structural analog of α-KG, exists as two enantiomers: D-2-HG and L-2-HG. In normal physiology, these are present at low levels and cleared by specific dehydrogenases (e.g., D-2-hydroxyglutarate dehydrogenase, D2HGDH). However, gain-of-function mutations in IDH1 or IDH2 convert α-KG into D-2-HG, leading to its pathological accumulation in various cancers such as acute myeloid leukemia (AML), glioma, chondrosarcoma, and cholangiocarcinoma [11, 12]. L-2-HG can accumulate under hypoxia or acidic conditions due to promiscuous activity of enzymes like lactate dehydrogenase (LDHA) and malate dehydrogenase (MDH), or via changes in redox balance. Overexpression of enzymes Such as 3-phosphoglycerate dehydrogenase (PHGDH), which alters the cellular NADH/NAD + ratio, has also been shown to indirectly increase L-2-HG levels [13–15].

Excess 2-HG inhibits α-KG–dependent dioxygenases, resulting in widespread DNA and histone hypermethylation, impaired differentiation, and immunosuppressive signaling within the tumor and surrounding immune cells.

### Succinate and fumarate

Succinate and fumarate are TCA cycle intermediates generated by succinate dehydrogenase (SDH) and fumarate hydratase (FH), respectively. Inherited or somatic mutations in SDH or FH result in the accumulation of these metabolites, which act as competitive inhibitors of α-KG–dependent enzymes. This inhibition leads to stabilization of hypoxia-inducible factors (HIFs), altered epigenetic landscapes, and inflammatory signaling pathways [16–19]. These oncometabolites are linked to cancers such as renal cell carcinoma, paraganglioma, pheochromocytoma, and hereditary leiomyomatosis and renal cell carcinoma (HLRCC), and contribute to immune evasion by modulating the activity of macrophages and T cells.

### Itaconate

Itaconate is derived from the TCA intermediate cis-aconitate and is produced in immune cells, especially Macrophages, through the activity of aconitate decarboxylase 1 (ACOD1/Irg1) [20, 21]. Although long considered restricted to immune cells, emerging evidence now indicates that tumor cells themselves can generate itaconate, adding complexity to its role as a key immunoregulatory metabolite in the tumor microenvironment [22]. In response to inflammatory stimuli such as lipopolysaccharide (LPS), itaconate accumulates in macrophages and modulates immune signaling by inhibiting SDH, activating NRF2, and regulating redox balance. Itaconate can either suppress or promote tumor progression depending on context, by influencing macrophage polarization, dendritic cell function, and T cell activity [23]. Its emerging role in cancer immunometabolism makes it a potential target for therapeutic modulation.

## Impact of oncometabolites on cancer progression

**Fig. 2:**
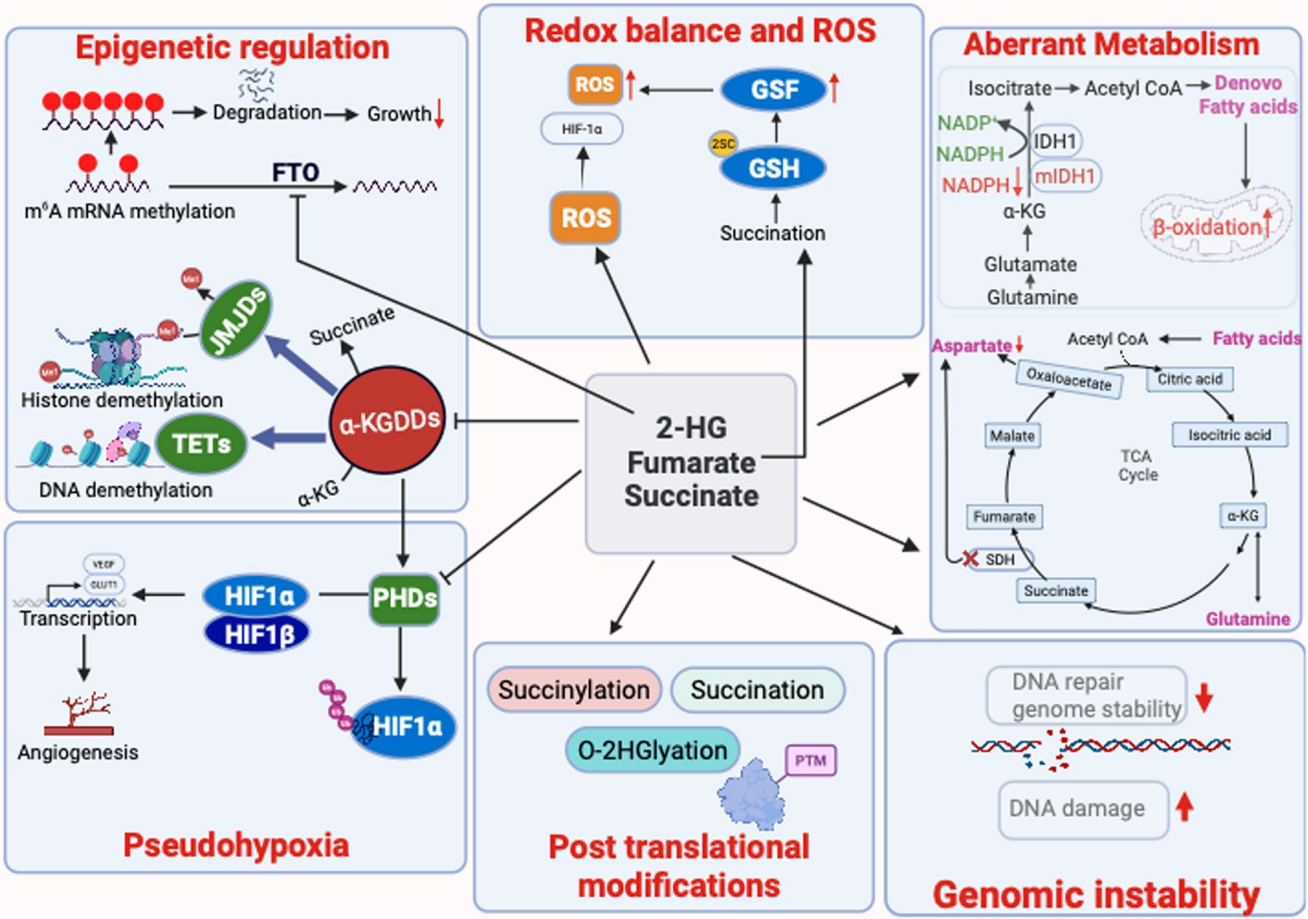
Oncometabolite-driven epigenetic and metabolic dysregulation in cancer. Oncometabolites Such as 2-HG, succinate, and fumarate, which accumulate due to mutations in IDH1/2, SDH, or FH, disrupt multiple cellular pathways. These metabolites competitively inhibit α-ketoglutarate–dependent dioxygenases (α-KGDDs), including TET DNA demethylases, histone demethylases, and the RNA demethylase FTO. This leads to widespread DNA and histone hypermethylation and increased m⁶A RNA methylation, altering gene expression and blocking cell differentiation. They also induce pseudohypoxia by inhibiting prolyl hydroxylases, stabilizing HIF-1α, and activating angiogenesis and glycolysis genes (e.g., VEGF, GLUT1). Redox balance is disrupted through multiple mechanisms: succinate enhances ROS via reverse electron transport; D-2-HG depletes NADPH and glutamate, impairing glutathione synthesis; and fumarate modifies GSH via Succination, forming 2-succinyl-cysteine (2SC). Tumor cells rewire their metabolism, shifting toward fatty acid β-oxidation and glutaminolysis to compensate for impaired TCA cycle activity and reduced aspartate synthesis. Mutant IDH1 tumors rely on pyruvate carboxylase and show lipid depletion. Additionally, oncometabolites induce post-translational modifications, including succinylation, succination, and O-2HGylation, which alter protein function, DNA repair, and cytoskeletal regulation. Together, these effects promote tumor progression and create therapeutic vulnerabilities

TCA cycle-derived oncometabolites can profoundly influence tumor cell behavior and progression. In this section, we explore how these metabolites modulate gene expression, redox balance, cellular metabolism, and genomic stability, contributing to cancer development (Fig. 2).

### Epigenetic effects of oncometabolites

Epigenetic modifications, such as DNA methylation, histone modifications, and chromatin remodeling, play a central role in regulating gene expression without altering the underlying DNA sequence. These processes are regulated by a family of α-KG-dependent dioxygenases, including TET enzymes, which catalyze DNA demethylation, and Jumonji C domain–containing histone demethylases. Oncometabolites Such as 2-HG, succinate, and fumarate interfere with these enzymes by mimicking α-KG and acting as competitive inhibitors [7, 24, 25].

D-2-HG, produced by mutant IDH1/2, accumulates to millimolar levels in certain cancers, including glioma, AML, and cholangiocarcinoma. Elevation of D-2-HG potently inhibits DNA demethylases and histone demethylases, leading to global DNA hypermethylation, histone methylation (e.g., H3K9me3, H3K27me3), and a repressive chromatin state that impairs cell differentiation and promotes tumorigenesis [7, 24, 26, 27]. In IDH1/2-mutant tumors, this hypermethylation is often observed as a CpG island methylator phenotype (CIMP), contributing to the silencing of tumor suppressor genes and lineage-specifying transcription factors [27, 28].

L-2-HG, which can accumulate under hypoxic or acidic conditions, also inhibits the same family of α-KG-dependent enzymes [14], but may induce distinct epigenetic changes and affect different sets of genes. L-2-HG has been implicated in maintaining stemness and delaying differentiation through similar inhibitory effects on demethylases [29, 30].

The accumulation of succinate and fumarate also has significant epigenetic consequences in tumorigenesis. Both are structurally similar to α-KG and act as competitive inhibitors of α-KG-dependent dioxygenases (2-OGDDs) [25, 31]. These include the TET family and Jumonji domain–containing histone demethylases. Their inhibition promotes DNA and histone hypermethylation, leading to widespread gene expression changes. For instance, succinate accumulation in SDH-deficient tumors can silence tumor suppressor genes via hypermethylation [31], while fumarate accumulation in FH-deficient tumors has been linked to silencing of neuroendocrine differentiation genes [32].

Beyond DNA and histone methylation, 2-HG also affects RNA epigenetics. By inhibiting the RNA demethylase FTO, 2-HG leads to increased N6-methyladenosine (m6A) RNA methylation, impacting mRNA stability, splicing, and translation [33].

In addition to the well-established accumulation of the oncometabolite D-2-HG, IDH mutations have also been shown to alter other metabolic intermediates. For example, comparative metabolomic analyses of IDH1-mutant gliomas revealed a significant increase in intracellular isocitrate levels, reflecting impaired oxidative decarboxylation of isocitrate to α-ketoglutarate and a shift in metabolic fluxes [34]. Biochemical studies further demonstrated that IDH mutations compromise not only the forward oxidative activity but also the reverse reductive carboxylation reaction, which normally contributes to citrate and acetyl-CoA production under hypoxia or mitochondrial dysfunction [35]. Since acetyl-CoA serves as the obligate donor for histone acetylation, fluctuations in its intracellular pool can directly impact chromatin accessibility and transcriptional programs [36]. Consistently, perturbations in acetyl-CoA metabolism have been linked to broad changes in histone acetylation and transcriptional reprogramming in multiple cancer contexts [37]. Therefore, changes in acetyl-CoA availability downstream of IDH mutation May represent an additional layer of epigenetic dysregulation beyond 2-HG–mediated inhibition of dioxygenases. Moreover, mitochondrial deacetylases Such as Silent Information Regulator 3 (SIRT3) provide another level of control by regulating the acetylation status of IDH2 and other metabolic enzymes [38, 39]. Loss of SIRT3 activity has been associated with hyperacetylation of IDH2, impaired enzymatic function, and enhanced tumorigenesis, underscoring how mitochondrial acetylation balance integrates metabolic and epigenetic reprogramming. Together, these findings Suggest that metabolic rewiring in IDH-mutant cells extends beyond 2-HG to encompass altered isocitrate and acetyl-CoA metabolism, with important implications for chromatin regulation and cancer biology.

These widespread epigenetic disruptions affect cellular plasticity, differentiation, and immune evasion and are often reversible upon inhibition of mutant IDH enzymes or metabolic reprogramming. As such, targeting epigenetic vulnerabilities induced by oncometabolites has become a major focus in cancer therapy.

### Effects on ROS

Oncometabolites, including 2-HG, succinate, and fumarate, disrupt redox balance in cancer cells and lead to the accumulation of reactive oxygen species (ROS). Succinate, which often builds up due to mutations in SDH, promotes reverse electron transport at mitochondrial complex I and increases ROS production [40, 41]. This oxidative stress stabilizes HIF-1α, supporting tumor growth [42]. However, ROS plays a dual role [43]: at moderate levels, it can promote cancer progression, while excessive ROS may damage DNA and proteins, induce cell death, or enhance the effectiveness of chemotherapy and radiotherapy. As a result, the impact of ROS depends heavily on its level and the tumor context.

Both enantiomers of 2-HG can increase oxidative stress but through distinct mechanisms. D-2-HG is produced by neomorphic activity of mutant IDH1 or IDH2 enzymes in several cancers. One key effect of D-2-HG is the consumption of NADPH during its synthesis from α-KG. Since NADPH is essential for regenerating glutathione (GSH), its depletion weakens cellular antioxidant defenses [44, 45]. D-2-HG also inhibits mitochondrial complex IV, further impairing electron transport and increasing ROS generation. Additionally, it lowers intracellular glutamate levels, another precursor for GSH synthesis, compounding the redox imbalance. Together, these effects lead to increased ROS that can contribute to DNA damage and oncogenic signaling, but may also sensitize cells to oxidative therapies depending on the context [45]. L-2-HG similarly disrupts redox homeostasis. Although its biology is less well understood, L-2-HG has been shown to increase mitochondrial ROS and lower GSH levels in vitro, suggesting it acts through similar oxidative mechanisms [46]. Both enantiomers thus shift the tumor environment toward oxidative stress, with context-dependent consequences for tumor progression or therapeutic vulnerability.

Fumarate contributes to ROS elevation through a distinct mechanism. It covalently modifies glutathione via cysteine succination, forming S-(2-succinyl)-cysteine (2SC), which reduces the availability of reduced glutathione (GSH) and weakens the cell’s antioxidant defense [47]. In addition, fumarate accumulation induces reductive stress and reroutes glucose metabolism toward the oxidative branch of the pentose phosphate pathway (PPP), creating a metabolic dependency that renders FH-deficient cells selectively vulnerable to PPP inhibition [48].

### Aberrant metabolism driven by oncometabolites

The buildup of oncometabolites alters normal metabolic pathways and supports cancer progression by reprogramming how tumor cells generate energy and biosynthetic precursors. This metabolic shift not only fuels proliferation but also supports adaptation to stress, invasiveness, and resistance to therapy.

Aspartate metabolism plays a central role in rapidly dividing cancer cells. Aspartate supplies nitrogen for purine and pyrimidine synthesis, making it essential for DNA and RNA production. It also supports redox homeostasis through the malate-aspartate shuttle, which helps maintain NAD⁺/NADH balance during glycolysis, especially under hypoxia [49–51]. Aspartate is typically produced in mitochondria via the enzyme glutamate oxaloacetate transaminase 2 (GOT2), which uses glutamate and the TCA cycle intermediate oxaloacetate [52]. Inhibition of glutaminase-1 (GLS1) in VHL-deficient renal cancers reduces aspartate synthesis and slows tumor growth [53]. Similarly, loss of SDH disrupts aspartate production. To compensate, cancer cells upregulate pyruvate carboxylase (PC) to convert glucose-derived pyruvate into oxaloacetate, enabling continued aspartate synthesis despite a dysfunctional TCA cycle [54].

Glutamine metabolism is another critical axis in cancer. Tumor cells show high glutamine uptake, termed "glutamine addiction", to support both energy production and biosynthesis [55]. Glutamine is converted into α-ketoglutarate (α-KG), feeding into the TCA cycle to sustain mitochondrial function [56]. It also serves as a substrate for glutathione (GSH) synthesis, maintaining antioxidant defenses. The balance between glutamate and GSH within mitochondria is crucial for managing oxidative stress and ensuring tumor cell survival under metabolic pressure [57].

Fatty acid metabolism contributes to tumor bioenergetics and biomass. Through β-oxidation, fatty acids are broken down into acetyl-CoA, which fuels the TCA cycle and supports ATP production. Acetyl-CoA also serves as a substrate for lipid biosynthesis, a process driven by enzymes such as ATP citrate lyase (ACLY), acetyl-CoA carboxylase (ACC), and fatty acid synthase (FAS). These enzymes are frequently overexpressed in cancers including lung, breast, liver, and prostate, and their activity is associated with tumor aggressiveness and poor prognosis [58]. Recent studies have revealed that mutant IDH1 tumors exhibit defective reductive carboxylation, sustained NADPH depletion, and a compensatory increase in fatty acid β-oxidation, leading to a specific reliance on ACC1 for both lipid synthesis and energy production. This metabolic rewiring distinguishes mIDH1 from mIDH2 tumors and contributes to mIDH1-specific perturbations in lipid profiles, including depletion of monoacylglycerides and lysophospholipids [59].

Both glycolysis and oxidative metabolism are essential for tumor cell growth, with the TCA cycle serving as a central hub that integrates these pathways to support energy production and biosynthesis. Cancer cells have been reported to exhibit metabolic plasticity, switching between aerobic processes and oxidative phosphorylation or performing both simultaneously. Therefore, understanding the TCA cycle and its components, as well as their interactions with other altered metabolic pathways, is crucial for comprehending tumors and their microenvironments for better therapeutic interventions.

### Pseudohypoxia

Cancer cells frequently adapt their metabolism to thrive in hostile environments. One such adaptation is pseudohypoxia, a condition in which cells activate hypoxia-associated pathways despite having normal oxygen levels. Although true hypoxia (low oxygen) is common in solid tumors and promotes aggressive phenotypes, pseudohypoxia mimics its effects, supporting tumor growth, survival, and immune evasion [60, 61]. This phenomenon has been reported in renal cell carcinoma, prostate cancer, lung adenocarcinoma, hepatocellular carcinoma, and pheochromocytomas/paragangliomas (PPGLs) [62–66]. It also reshapes immune interactions by fostering an immunosuppressive tumor microenvironment [62, 67–69].

Pseudohypoxia is driven by the inappropriate stabilization of HIFs, particularly HIF-1α and HIF-2α. Under normal oxygen conditions, prolyl hydroxylases (PHDs) hydroxylate HIFα subunits, marking them for degradation by the von Hippel–Lindau (VHL) E3 ubiquitin ligase. Hypoxia inhibits PHDs, allowing HIFα to accumulate, translocate to the nucleus, and activate transcription with cofactors like p300/CBP [70]. In cancer, however, HIFs can become stabilized even in normoxia due to oncometabolite accumulation. This triggers gene expression programs associated with angiogenesis, epithelial–mesenchymal transition (EMT), and stemness, factors that enhance malignancy [71, 72].

Mutations in TCA cycle enzymes such as SDH, FH, IDH, and MDH2 result in the buildup of oncometabolites like Succinate, Fumarate, 2-HG, and malate. These metabolites inhibit α-KG–dependent PHDs, stabilizing HIFs and initiating pseudohypoxic signaling [60, 73–75]. In contrast, elevated α-KG levels can reverse these effects and suppress tumor growth [76, 77]. HIF activity also shifts metabolism by reducing citrate levels relative to α-KG, thereby promoting reductive carboxylation [78]. A recent study showed that glucose restriction in lung adenocarcinoma decreases α-KG levels and downregulates histone demethylases, leading to hypermethylation and enhanced HIF stabilization via EZH2-mediated repression of PHD3 [65]. These findings highlight the potential risks of targeting glucose metabolism in cancer therapy, as such interventions might inadvertently promote more aggressive tumor behaviour.

Beyond PHD inhibition, elevated level of fumarate promotes pseudohypoxia through alternative signaling. It activates nuclear factor kappa-light-chain-enhancer of activated B cells (NF-κB) via Tank-binding kinase 1 (TBK1), increasing HIF-1α transcription independently of the VHL pathway [79]. Blocking the TBK1–p65 axis reduces HIF-1α levels and decreases invasiveness in FH-deficient renal cell carcinoma. Fumarate can also activate NRF2 by succinating KEAP1, and NRF2, in turn, binds HIF-1α and prevents its hydroxylation by PHD2, enhancing HIF stability [64, 80]. Together, these mechanisms illustrate how fumarate reinforces pseudohypoxia and tumor aggressiveness through multiple converging pathways.

### Oncometabolites and genomic instability

Genomic instability, marked by accumulated DNA damage and impaired repair mechanisms, is a key driver of cancer evolution and heterogeneity. Rather than being a passive consequence of tumorigenesis, it actively fuels malignant progression. Two major forms of genomic instability are commonly recognized: chromosomal instability (CIN), involving structural or numerical chromosomal abnormalities, and microsatellite instability (MSI), characterized by defects in short tandem repeat sequences due to impaired mismatch repair. While germline mutations in DNA repair genes explain instability in hereditary cancers, sporadic tumors often acquire instability through more subtle mechanisms, including metabolic and epigenetic disruption [81, 82].

Key tumor suppressors such as ATM (Ataxia-Telangiectasia Mutated) and p53 serve as central regulators of the DNA damage response, coordinating cell cycle arrest, DNA repair, senescence, or apoptosis to maintain genomic integrity [83]. However, emerging evidence Suggests that oncometabolites, particularly 2-HG, succinate, and fumarate, interfere with these protective pathways.

These metabolites impair homologous recombination (HR), a major mechanism for repairing DNA double-strand breaks. Their accumulation inhibits α-ketoglutarate–dependent dioxygenases, including histone demethylases such as KDM4B. This leads to abnormal accumulation of H3K9me3 at DNA break sites, which in turn disrupts the recruitment of TIP60 and ATM, two proteins essential for initiating HR-mediated repair [84]. Consequently, cells become more reliant on error-prone repair pathways, contributing to mutagenesis and tumor progression. Supplementing cells with exogenous α-KG can reverse these effects, restoring histone demethylation and reactivating HR repair [85].

In addition to intrinsic effects within tumor cells, oncometabolites may affect the genomic stability of neighboring stromal cells, including immune cells, fibroblasts, and endothelial cells. These cells are increasingly recognized to harbor somatic mutations in the tumor microenvironment [86]. One possibility is that exposure to high levels of secreted oncometabolites, Such as 2-HG, may induce oxidative stress or epigenetic alterations in these non-epithelial cells, contributing to their transformation or dysfunction.

### Post-translational modification by oncometabolites

Post-translational modifications (PTMs) are critical regulators of protein activity, stability, and localization. In cancer, accumulating evidence shows that metabolites, especially those altered in the TCA cycle, can drive aberrant PTMs, contributing directly to tumor progression. These metabolite-induced modifications link cellular metabolism to oncogenic signaling, genome instability, and therapy resistance [87–90].

Succinylation, a reversible PTM involving the addition of succinyl groups to lysine residues, is driven by increased levels of succinyl-CoA. This modification alters protein conformation and charge, influencing pathways that regulate metabolism, mitochondrial function, and DNA repair [89]. For example, in pancreatic ductal adenocarcinoma (PDAC), SUCLA2-dependent succinylation of glutaminase (GLS) enhances NADPH and glutathione (GSH) production, improving redox balance and promoting survival. In hepatocellular carcinoma (HCC), OXCT1 functions as a lysine Succinyl transferase, modifying LACTB at lysine 284 to enhance mitochondrial membrane potential and support tumor growth [91].

Succination, distinct from succinylation, refers to the irreversible modification, occurs when fumarate, a metabolite elevated in FH-deficient tumors, covalently binds cysteine residues to form S-(2-succinyl)-cysteine (2SC). This alters protein function by inactivating redox enzymes and disrupting DNA repair machinery, contributing to oxidative stress and genomic instability [92].

O-2-Hydroxyglutarylation (O-2HGylation) is a newly discovered protein modification caused by the oncometabolites D-2-HG and L-2-HG [90]. These metabolites can covalently modify serine, threonine, and tyrosine residues in a stereospecific manner, altering protein structure and function. For example, D-2-HG selectively modifies MRCKA (myotonic dystrophy kinase-related Cdc42-binding kinase alpha), a serine/threonine kinase involved in regulating cytoskeletal organization, actomyosin contractility, and cell motility. O-2HGylation reduces cytoskeletal tension and directional migration by blocking MRCKA-mediated phosphorylation of myosin light chain (MLC), thereby promoting features associated with cancer cell invasion and metastasis. In contrast, L-2-HG modifies SLK (STE20-like kinase), another serine/threonine kinase that regulates cell adhesion, cytoskeletal remodeling, and stress-induced signaling pathways. O-2HGylation of SLK reduces its catalytic activity, leading to diminished phosphorylation of Substrates involved in focal adhesion dynamics and cell Survival. This covalent modification provides a direct mechanism by which 2-HG influences protein activity, adding to its well-characterized roles in epigenetic reprogramming.

While O-2HGylation has been studied primarily in tumor cells, it remains unclear whether 2-HG secreted into the tumor microenvironment can be taken up by neighboring immune or stromal cells and induce similar modifications. If so, this mechanism could represent a new layer of tumor-driven immune modulation. In addition, no enzymes have yet been identified that write, erase, or recognize O-2HGylation, leaving its regulation and functional consequences largely unexplored. These open questions highlight the need for further research into the broader biological significance and therapeutic potential of this novel post-translational modification.

Together, succinylation, succination, and O-2HGylation illustrate how oncometabolites can directly reprogram protein function and regulatory networks in cancer. These PTMs provide critical mechanistic insight into how metabolism shapes tumor behavior and offer promising opportunities for biomarker discovery and therapeutic intervention in precision oncology.

## Impact of oncometabolites on tumor microenvironment

**Fig. 3:**
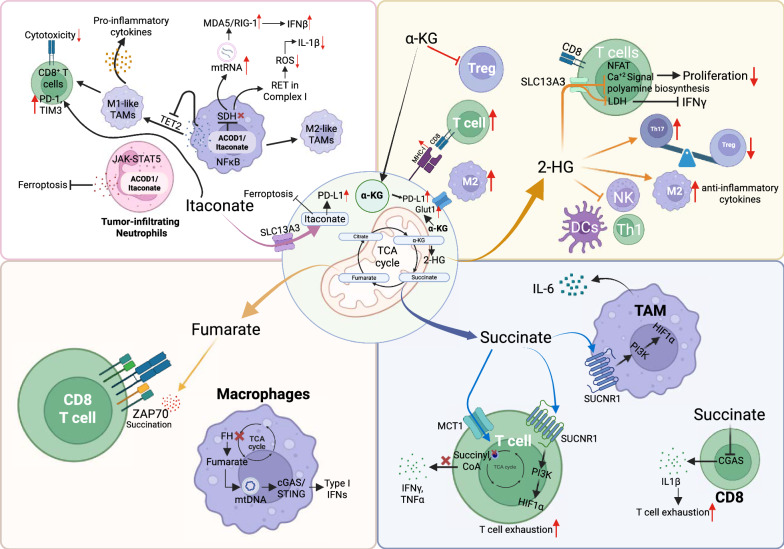
Immunomodulatory roles of TCA cycle oncometabolites in the tumor microenvironment. TCA-derived metabolites regulate immune cell behavior in cancer. α-KG enhances antigen presentation and CD8⁺ T cell Function through epigenetic remodeling. In contrast, 2-HG impairs T cell metabolism and activation, silences innate immune genes like cGAS in tumor and myeloid cells, and promotes M2 macrophage polarization. Succinate enhances immunosuppression by stabilizing HIF-1α, activating SUCNR1 signaling, and impairing T cell function through ROS and metabolic disruption. Fumarate succinates and inactivates TCR signaling proteins like ZAP70, contributing to T cell dysfunction. Itaconate, produced by myeloid cells, has dual roles: it can dampen inflammation and promote T cell exhaustion or, under specific conditions, activate antiviral signaling pathways. In tumor cells, imported itaconate stabilizes PD-L1 and protects against ferroptosis, promoting immune evasion. These combined effects create an immune-suppressive microenvironment that hinders anti-tumor immunity

Beyond their roles within cancer cells, oncometabolites also profoundly influence the surrounding tumor microenvironment. These metabolites act not only as intracellular regulators but also as paracrine signals that reshape the behavior of immune and stromal cells. By altering gene expression, metabolism, and signaling pathways in T cells, macrophages, dendritic cells, endothelial cells, and fibroblasts, oncometabolites contribute to immune suppression, impaired antigen presentation, and resistance to immunotherapy. In this section, we examine how specific TCA-related metabolites modulate the immune landscape and tumor-host interactions (Fig. 3).

### α-Ketoglutarate (tumor-suppressive metabolite)

α-KG, a key TCA cycle metabolite, has emerged as a central regulator of immune function in the tumor microenvironment. It shapes immune responses by influencing cell fate, epigenetic remodeling, and metabolic signaling across multiple immune cell types.

In CD4⁺ T cells, exogenous α-KG limits differentiation into regulatory T cells (Tregs) and promotes inflammatory cytokine expression. This is linked to metabolic rewiring, as α-KG feeds fatty acid synthesis and alters lipid composition, including increased triacylglycerol (TAG) storage [93]. In renal cell carcinoma, low α-KG levels correlate with immune evasion. Supplementation restores β2-microglobulin (B2M) expression by promoting H3K4 demethylation at its promoter, enhancing MHC class I expression and antigen presentation to cytotoxic T cells [94]. α-KG also affects dendritic cells by modulating redox balance, which influences their ability to activate T cells [95].

In tumors, α-KG cooperates with NAD⁺ (maintained by NAMPT) to support TET1-mediated demethylation of the IRF1 promoter, enabling IFNγ-driven PD-L1 expression. This epigenetic loop contributes to immune evasion but also makes tumors more responsive to PD-L1 blockade [96].

α-KG can be produced not only through the TCA cycle but also via glutaminolysis, which alters the α-KG to succinate ratio and promotes anti-inflammatory (M2-like) polarization. α-KG activates the histone demethylase Jmjd3, removing repressive marks on M2-associated genes and enabling their expression [97]. It also plays a role in dampening inflammation by contributing to endotoxin tolerance following pro-inflammatory (M1) activation [97]. Further supporting this, IL-4 stimulation activates a mitochondrial signalling axis involving SENP1, SIRT3, and GLUD1. SENP1 de-SUMOylates and activates SIRT3, which in turn deacetylates GLUD1 to enhance α-KG production from glutamate. This increase in α-KG reinforces M2 polarization. Notably, this process occurs independently of fatty acid oxidation, revealing an alternative glutaminolysis-driven mechanism for promoting anti-inflammatory macrophage states [98].

### 2-Hydroxyglutarate

Defective immune surveillance is a hallmark of IDH1-mutant (mIDH1) tumors [99–101]. These tumors typically exhibit an immunologically “cold” microenvironment, characterized by low infiltration of cytotoxic T cells and impaired interferon responses [99, 101–104]. Emerging studies have begun to unravel how mutant IDH1 and its oncometabolite, D-2-HG, shape the TME to evade immune attack.

D-2-HG drives widespread DNA hypermethylation, leading to the silencing of key immune-related genes. In several IDH1-mutant cancers, including intrahepatic cholangiocarcinoma (ICC), glioma, melanoma, chondrosarcoma, and prostate cancer, this includes the epigenetic repression of cGAS, a DNA sensor critical for activating the STING pathway and type I interferon signalling [104]. Silencing of cGAS leads to reduced recruitment of CD8⁺ T cells, further weakening antitumor immunity. However, it is not observed in leukaemia. D-2-HG also impairs IFN-γ signalling by inhibiting the DNA demethylase TET2, which is necessary for activating interferon-stimulated genes [99]. Moreover, in gliomas, additional suppression of antiviral genes IRF3 and IRF7 has been attributed to D-2-HG-enhanced DNMT1 binding at their promoters, further reducing type I interferon responses [105]. Interestingly, while ATRX loss is associated with increased immune activation in glioma, co-occurring IDH1 mutations can mask this effect, a suppression reversible with mutant IDH1 inhibition [106].

Beyond epigenetic silencing, mIDH1 tumors also impair T cell trafficking. The chemokine CXCL10, which recruits CD8⁺ T cells, is downregulated due to suppressed STAT1 signalling, leading to fewer T cells in the TME [107]. More broadly, reduced leukocyte chemotaxis, including diminished infiltration of T cells, macrophages, monocytes, and neutrophils, has been observed in both human and mouse models of mIDH1 glioma [102]. Additionally, glioma stem-like cells with IDH1 mutations secrete granulocyte colony-stimulating factor (G-CSF), reprogramming tumor-infiltrating neutrophils into non-suppressive states, which further alters immune dynamics [108].

D-2-HG also acts through paracrine mechanisms. It is secreted by tumor cells and taken up by T cells via the SLC13A3 transporter. Once inside, D-2-HG impairs NFAT transcriptional activity, calcium signalling, and polyamine biosynthesis, leading to reduced T cell proliferation, function, and memory formation [100]. It also suppresses CD8⁺ T cell metabolism by disrupting lactate dehydrogenase (LDH)-dependent pathways, diminishing cytotoxic function and IFN-γ production [103]. At the same time, D-2-HG limits the differentiation of regulatory T cells (Tregs) by epigenetically silencing the FOXP3 locus, instead skewing T cells toward a Th17 phenotype [109, 110]. This dual effect, blocking both effector and regulatory T cell programs, underscores the complex immunoregulatory role of D-2-HG in the TME.

D-2-HG also reprograms myeloid cells, including dendritic cells (DCs) and macrophages, which play central roles in coordinating antitumor immunity. In DCs, D-2-HG impairs maturation and function by downregulating CD83 and suppressing the secretion of interleukin-12 (IL-12), a key cytokine for activating natural killer (NK) cells and promoting Th1 differentiation. This limits the ability of DCs to prime effective T cell responses and present tumor antigens, thereby weakening overall immune surveillance [111]. Macrophages in the tumor microenvironment take up D-2-HG through the SLC13A3 transporter, which promotes their polarization toward an M2-like, immunosuppressive phenotype. These macrophages secrete anti-inflammatory cytokines and pro-angiogenic factors that support tumor progression. Mechanistically, D-2-HG activates tryptophan 2,3-dioxygenase, enhancing kynurenine production and activating the aryl hydrocarbon receptor (AHR), further reinforcing immune suppression. This myeloid cell reprogramming contributes to an immunosuppressive niche that limits immune-mediated tumor clearance [112].

D-2-HG also promotes angiogenesis through its effects on endothelial cells. These cells express the SLC1A1 transporter, which facilitates D-2-HG uptake and mitochondrial accumulation. Within mitochondria, D-2-HG enhances Na⁺/Ca^2^⁺ exchange and activates the respiratory chain, promoting endothelial cell migration. This leads to the formation of abnormal, leaky vasculature that not only supports tumor growth but also impairs immune cell infiltration, creating both physical and functional barriers to antitumor immunity [113].

The other enantiomer of 2-HG, L-2-HG, accumulates in certain tumors, such as clear cell renal carcinoma and pancreatic cancer [30, 114], and plays a distinct role in modulating immune responses. In vivo treatment with its cell-permeable form, octyl ester L-2-HG (OE-L-2-HG), enhanced tumor infiltration and antitumor activity in multiple adoptive T cell transfer models, supporting its potential in cancer immunotherapy [115]. RNA-seq analyses revealed divergent transcriptional programs in CD8⁺ T cells treated with OE-L-2-HG versus OE-D-2-HG, reflecting opposing effects on T cell differentiation. OE-L-2-HG promoted early memory-like CD8⁺ T cells (CD62L⁺/CD45RO⁺ and CCR7⁺/CD45RO⁺), while OE-D-2-HG drove a more terminally differentiated phenotype (CD62L⁻/CD45RO⁺) [115]. L-2-HG also reprograms macrophages, enhancing their antigen presentation capacity and improving CD8⁺ T cell activation. This effect improved responses to anti–PD-1 therapy and may help overcome resistance. Furthermore, blocking L2HGDH, the enzyme that degrades L-2-HG, reprograms macrophages into an antitumoral state, boosting T cell–mediated immunity [116].

Together, these findings emphasize the opposing immunologic roles of D-2-HG and L-2-HG. By broadly reprogramming T cells, dendritic cells, macrophages, and endothelial cells, D-2-HG promotes immune evasion and tumor progression. Targeting D-2-HG and its downstream effects offers a promising strategy to restore immune function in IDH1-mutant cancers.

### Succinate

Succinate accumulation, driven by either cancer cell secretion or metabolic dysfunction within immune cells, can significantly alter the functional properties of T cells and macrophages, acting as an intracellular and extracellular signalling molecule to modulate immune cell activity. Elevated levels of Succinate and the expression of Succinic acid receptor 1 (SUCNR1 or GPR91) have been associated with significant alterations in the immune cell functions in the ovarian cancer microenvironment [117, 118]. Specifically, succinate promoted a pro-tumorigenic phenotype in tumor-associated macrophages (TAMs), contributing to immune suppression and tumor growth [118, 119]. TAMs support tumor progression by secreting pro-migratory cytokines like IL-6 and induce tumor metastasis through PI3K/AKT and HIF-1a signalling [120]. Pharmacological inhibition of SDH using dimethyl malonate (DMM) enhanced M2 macrophage polarization through mitochondrial ROS-dependent activation of the STAT6 pathway [74]. DMM treatment decreased pro-inflammatory cytokine IL-1β levels and increased anti-inflammatory cytokine IL-10 levels in lipopolysaccharide (LPS)-activated M1 macrophages and increased the expression of M2-associated genes such as *Arg1*, *Ym1*, and *Mrc1* in IL-4-activated macrophages [121].​

In T cells, tumor-associated concentrations of succinate impair effector functions. Human CD4⁺ and CD8⁺ T cells exposed to high levels of succinate exhibit reduced degranulation and cytokine secretion, including interferon-γ (IFN-γ) [122]. ​ Succinate is taken up by T cells partly via monocarboxylate transporter 1 (MCT1), which inhibits succinyl coenzyme A synthetase activity and impairs glucose flux through the TCA cycle. ​ The resulting metabolic disruption reduces expression of activation markers (CD25, CD69) and diminishes T cell effector function [122]. T cells deficient in SDH exhibited impaired proliferation and increased cell death due to disrupted TCA cycle flux, leading to insufficient nucleoside biosynthesis [123]. Succinate accumulation can also promote immune evasion through effects on PD-L1. Increased chondroitin Sulfate synthase 1 (CHSY1) activity leads to succinate accumulation and activation of the PI3K/AKT/HIF-1α signalling axis, thereby upregulating PD-L1 and promoting CD8⁺ T cell exhaustion [124].

In contrast, other studies demonstrated opposing effects of Succinate and SDH in T cells. In one study SDH-deficient T cells exhibited a pro-inflammatory gene signature, with increased differentiation into T Helper 1 (Th1) and T Helper 17 (Th17) lineages [123]. This was associated with an elevated succinate/α-ketoglutarate ratio, which altered chromatin accessibility and transcriptional patterns, notably upregulating the transcription factor PR domain zinc finger protein 1 (PRDM1) [123]. In a recent study, succinate was seen to sustain the stem-like phenotype in CD8^+^ T cells, which is linked to long-term persistence and durability of antitumor responses. This is attributed to epigenetic remodelling induced by succinate. T cells, including CART cells, conditioned with succinate and adoptively transferred into mice, showed persistence and more tumor control. Succinate exposure promotes mitochondrial health in CD8⁺ T cells through BNIP3-mediated mitophagy, a process that removes damaged mitochondria, thus renewing and improving mitochondrial function [125]. These contrasting results may reflect differences in cellular context, genetic models, or pharmacological interventions, and underscore the multifaceted roles of metabolic intermediates not only as energy substrates but also as regulators of gene expression and immune responses. In addition to these intrinsic mechanisms, tumor-associated succinate can also arise from extrinsic sources, such as the gut microbiota. For instance, in colorectal cancer, succinate produced by *Fusobacterium nucleatum* inhibits the cGAS–interferon-β pathway, reducing CD8⁺ T cell infiltration and promoting resistance to PD-1 checkpoint blockade [126].

### Fumarate

Fumarate accumulation due to FH deficiency in tumor cells results in epigenetic alterations such as DNA hypermethylation [32]. This epigenetic reprogramming can promote tumor progression and has been linked to immune evasion mechanisms [19, 127]. FH-deficient renal cell carcinoma (RCC) tumors exhibit high PD-L1 expression on tumor cells, along with elevated PD-1 expression on infiltrating immune cells [128]. Elevated fumarate leads to a suppressive tumor microenvironment by disrupting the T cell receptor (TCR) signalling. Specifically, fumarate accumulation leads to covalent modification of ZAP70 by a process called succination, in which fumarate reacts with cysteine residues. This post-translational modification impairs ZAP70 kinase activity, disrupting TCR signalling and thereby reducing CD8⁺ T cell activation and proliferation [129]​. Fumarate also induces epigenetic changes in CD8⁺ T cells, such as histone modifications, contributing to their dysfunctional state [130]​. Beyond effects on T cells, fumarate also reprograms macrophage responses. Gene expression analysis showed that fumarate affects IL-10 and TNF pathways in activated macrophages, helping control inflammation [131]. These immunomodulatory roles extend further into innate immune activation. Elevated fumarate levels trigger the release of mitochondrial DNA–containing vesicles into the cytosol, which in turn activate the cGAS–STING pathway and promote type I interferon production and other inflammatory cytokines [132].

### Itaconate

High expression of ACOD1 is reported in neutrophils in infection and tumor models [133] [134]. In tumor-infiltrating neutrophils (TINs), ACOD1 expression is driven by GM-CSF–JAK/STAT5–C/EBPβ signalling. ACOD1-mediated itaconate production activates NRF2, inducing antioxidant genes and conferring resistance to ferroptosis. ACOD1 deletion in neutrophils reduces TIN infiltration and lung metastasis, enhances antitumor T cell immunity, and improves responses to immune checkpoint blockade, without affecting primary tumor growth [134].​ ​

In macrophages, itaconate is mainly produced in response to inflammatory stimuli like LPS and within tumors [135, 136]. Itaconate inhibits SDH (complex II of the ETC), leading to succinate accumulation and modulation of the inflammatory response [135]. Irg1 knockout mice exhibit elevated IL-6, IL-12, and IL-1β levels, while exogenous itaconate suppresses these cytokines in vitro [9]. In contrast, another study showed the necessity of itaconate in promoting type I IFN responses. Both endogenous and exogenous itaconate inhibit SDH activity in macrophages.​ Inhibition of SDH leads to the release of mitochondria RNA (mtRNA) into the cytosol through the voltage-dependent anion Channel 1 (VDAC1).​ The released mtRNA, which activates cytosolic RNA sensors MDA5 and RIG-I, is essential for the induction of IFNβ [137]. Itaconate treatment resulted in enhanced expression of IFNβ and Cxcl10 [138]. Itaconate and its synthetic derivative 4-octyl Itaconate (OI) inhibit the phosphorylation of JAK1 and its downstream target STAT6, essential for the IL-4 signalling, thereby suppressing M2 polarization [139]. However, nasopharyngeal carcinoma (NPC) induces M2 macrophage polarization and increased itaconate production. TAM-derived itaconate suppresses CD8⁺ T cell cytotoxicity and phagocytic activity by inhibiting TET2, a DNA demethylase involved in immune regulation [140]. ​In hepatocellular carcinoma, itaconate promotes CD8⁺ T cell exhaustion by enhancing H3K4me3 histone modifications at the promoter region of the transcription factor Eomesodermin (EOMES), leading to PD-1 and TIM-3 expression [141].

Beyond its immune-modulatory roles, itaconate also exerts cell-intrinsic effects on tumor cells. Extracellular Itaconate, produced by activated macrophages, is taken up by the tumor cells through the SLC13A3 transporter. Once inside, itaconate alkylates PD-L1 at cysteine 272, preventing its ubiquitination and degradation, stabilizing PD-L1 levels on tumor cells and promoting resistance to immunotherapy [142]. SLC13A3 inhibition enhances anti-CTLA-4 efficacy and prolongs survival in mouse tumor models [142].

Additionally, internalized itaconate activates the NRF2-SLC7A11 antioxidant pathway, leading to resistance against ferroptosis, a form of regulated cell death in tumor cells [143]. Interestingly, tumor cells may also produce itaconate intrinsically, which paradoxically enhances tumor immunogenicity by increasing MHC-I expression and type I interferon signalling, resulting in higher CD8⁺ T cell infiltration [22].

Paralleling this duality, itaconate has also been shown to impair CD8⁺ T cell proliferation and cytokine production by limiting the biosynthesis of aspartate and serine/glycine. Mice lacking the Irg1 gene exhibited decreased tumor growth, reduced immunosuppressive activity of MDSCs, enhanced CD8⁺ T-cell-mediated anti-tumor immunity, and improved responses to anti-PD-1 therapy [144]. ​

Together, these findings suggest that targeting ACOD1 with small-molecule inhibitors may offer a promising therapeutic strategy, particularly when combined with immune checkpoint blockade.

## Therapeutic targeting of oncometabolite pathway

Targeting cancer metabolism has moved from concept to clinic, with oncometabolites now recognized as drivers of tumor progression and immune evasion. Mutant IDH inhibitors exemplify how reversing metabolite accumulation can yield clinical benefit. Building on this success, new therapies are being developed to disrupt metabolic vulnerabilities such as altered α-ketoglutarate signalling, glutamine metabolism, and itaconate regulation. This section highlights emerging and established strategies targeting oncometabolism across cancer types (Table 1).

**Table 1:** Therapeutic strategies targeting TCA cycle–derived oncometabolite pathways in cancer

Therapeutic strategy	Target/mechanism	Cancer types	Clinical efficacy	Developmental status
Mutant IDH inhibitors (Ivosidenib – IDH1, Enasidenib – IDH2)	Reduce 2-HG accumulation, restore differentiation	AML, MDS, Intrahepatic cholangiocarcinoma, glioma, chondrosarcoma	AML: improved OS (24.0 vs. 7.9 months, AGILE trial); MDS: ~ 39% response rate; Cholangiocarcinoma: improved PFS & OS (ClarIDHy trial); Glioma: strong 2HG suppression	FDA-approved for AML, MDS, cholangiocarcinoma; Glioma & chondrosarcoma in trials
Olutasidenib (IDH1 inhibitor)	Brain-penetrant, 2:1 stoichiometric binding	AML, glioma	Response rates: 56% (combo), 38% (mono)	FDA-approved (AML); ongoing trials for glioma
Vorasidenib (dual IDH1/2 inhibitor)	Brain-penetrant IDH1/2 inhibitor	Grade 2 IDH-mutant gliomas	PFS extended (27.7 vs. 11.1 months, INDIGO trial)	FDA-approved (2024)
Synthetic lethal strategies in mIDH1	Exploit metabolic/epigenetic vulnerabilities beyond 2HG	Glioma, AML, Solid tumors	NAMPT inhibition, DHODH inhibitors, GLS inhibitors, ACC1 targeting, PARP inhibitors show strong preclinical efficacy; Venetoclax effective in mIDH1 AML	Preclinical / early clinical trials
α-KG mimetics & modulators (DMKG, CPI-613/Devimistat)	Restore tumor-suppressive functions of α-KG; disrupt PDH/α-KGDH	TNBC, melanoma, AML, pancreatic cancer, biliary tract cancer	DMKG sensitizes tumors to PD-L1 blockade (preclinical); CPI-613 boosts PD-L1 response in melanoma; mixed trial results in AML/pancreatic cancer; BilT-04 trial showed 45% response in biliary cancer	CPI-613 in clinical trials (Phase II/III); DMKG preclinical
Glutaminase (GLS) inhibitors (BPTES, Telaglenastat/CB-839)	Block glutamine metabolism → reduce α-KG supply	RCC, TNBC, NSCLC, hematologic malignancies	RCC: improved PFS (ENTRATA trial); failed in CANTATA Phase II; modest activity in TNBC/NSCLC	Clinical trials (mixed results)
GDH inhibitors (EGCG, R162)	Block glutamate → α-KG conversion	Cholangiocarcinoma, glioma, HCC	Suppress tumor growth (preclinical); EGCG tested in CRC, ovarian, liver cancers	Preclinical & early clinical trials
Itaconate pathway modulators (DI, citraconate)	Modulate immunometabolite signaling (ACOD1/Irg1)	Colorectal cancer, immune-driven tumors	DI reduced inflammation & tumor growth in CRC models; citraconate activates NRF2–SLC7A11 axis (preclinical)	Preclinical

This table summarizes current therapeutic approaches directed at oncometabolite pathways, including their molecular targets, intended cancer types, clinical efficacy, and developmental status. Developmental status is categorized as preclinical (experimental studies in models), clinical trials (under investigation in human studies), mixed (evidence from both preclinical and clinical data), or FDA-approved (approved for clinical use)

### Targeting 2-HG in IDH-mutant cancers

The most clinically advanced example of targeting an oncometabolite is the inhibition of mutant IDH1 and IDH2 enzymes, which drive cancer through the aberrant production of 2-HG. Therapeutic efforts have focused on reducing 2-HG levels to restore cellular differentiation and improve patient outcomes, particularly in hematologic malignancies and select solid tumors.

Ivosidenib (AG-120, IDH1 inhibitor) and Enasidenib (AG-221, IDH2 inhibitor) have shown clinical efficacy in AML. In the Phase III AGILE trial (NCT03173248), Ivosidenib combined with azacitidine significantly improved overall survival compared to azacitidine alone (median overall Survival: 24.0 months vs. 7.9 months), establishing a new standard of care for IDH1-mutant AML [145, 146]. In relapsed/refractory myelodysplastic syndrome (MDS), Ivosidenib achieved a 38.9% response rate and a median Survival of 35.7 months in a Phase I study (NCT02074839) [147], with a Phase III frontline trial (PyramIDH, NCT06465953) currently underway..

Olutasidenib (FT-2102), a brain-penetrant, FDA-approved IDH1 inhibitor, demonstrated a 56% response rate in combination therapy and 38% as monotherapy, with durable responses in relapsed/refractory AML [148–150]. Its unique 2:1 stoichiometric binding may help overcome resistance seen with Ivosidenib.

Beyond haematological malignancies, in intrahepatic cholangiocarcinoma, the Phase III ClarIDHy trial demonstrated that Ivosidenib significantly improved progression-free survival and overall survival (10.3 vs. 7.5 months; 5.1 months after adjusting for crossover) [151, 152]. The trial also showed plasma 2-HG levels were reduced by up to 97% after one treatment cycle, reaching levels similar to healthy individuals [153, 154]. These reductions were observed even in patients with stable or progressive disease. Mechanistically, Ivosidenib promoted hepatocyte differentiation and suppressed biliary lineage markers, cell cycle progression, and AKT pathway activity [155].

For chondrosarcoma, Ivosidenib is under investigation in a Phase III trial (NCT06127407 – CHONQUER), following Phase I data showing 52% of patients achieved stable disease with a median PFS of 5.6 months [154].

Following early data showing Ivosidenib Suppressed tumor 2-HG in glioma [156], Vorasidenib, a dual IDH1/2 inhibitor with improved brain penetrance, was evaluated in a Phase I study (NCT03343197). Vorasidenib reduced intratumoral 2-HG by over 90%, and RNA-seq of treated tumors revealed induction of IFN-α and IFN-γ response signatures, along with increased expression of viral response genes such as OAS1, OAS2, IFITM1, and IFNB1, Suggesting that 2-HG suppression reactivates innate immune pathways These changes coincided with higher CD3⁺ and CD8⁺ T cell infiltration, implying that IDH inhibition may restore antitumor immunity [104, 157]. In the INDIGO Phase III trial (NCT04164901), Vorasidenib extended progression-free survival (27.7 vs. 11.1 months) in grade 2 IDH-mutant gliomas, leading to FDA approval in August 2024 despite manageable elevations in ALT, a marker of liver toxicity [158].

Similar immune activation was observed in mIDH1 intrahepatic cholangiocarcinoma. In a Phase I trial of Ivosidenib (NCT02073994), RNA-seq analysis of matched pre- and on-treatment samples (8–9 weeks of treatment) revealed upregulation of IFN-α and IFN-γ response genes, consistent with preclinical findings [104]. Preclinical studies in mIDH1-driven tumor models have shown that Ivosidenib restores IFN-γ sensitivity while inducing compensatory immune suppression, including PD-L1 upregulation, increased PD-1 and CTLA4 expression on CD8⁺ T cells, and Treg recruitment [99]. These mechanisms may limit the efficacy of PD-1 mono- or combo- therapy, whereas CTLA4 blockade synergizes with mIDH1 inhibition by depleting Tregs and enhancing cytotoxic T cell activity. Together, these results suggest that IDH inhibitors not only reduce oncometabolite burden but may also reshape the tumor immune microenvironment through restoration of interferon and viral mimicry pathways.

### Synthetic lethal strategies in mIDH1 cancers

While IDH1 inhibitors reduce 2-HG and restore differentiation, accumulating evidence shows that mIDH1 induces broader metabolic and epigenetic rewiring, creating unique vulnerabilities that persist even after 2-HG suppression. These synthetic lethal interactions offer promising therapeutic entry points, either as standalone strategies or in combination with IDH inhibitors.

One key vulnerability lies in NAD⁺ metabolism. mIDH1 gliomas downregulate NAPRT1, rendering cells dependent on the NAD⁺ salvage enzyme NAMPT. NAMPT inhibition selectively kills mIDH1 tumor cells and induces tumor regression in orthotopic glioma xenografts [159]. Another targetable liability is nucleotide metabolism. mIDH1 gliomas rely heavily on de novo pyrimidine synthesis due to impaired mitochondrial aspartate production, rendering them highly sensitive to DHODH inhibitors Such as BAY 2402234 in GEMMs and patient-derived models [160]. DHODH (dihydroorotate dehydrogenase) is a mitochondrial enzyme essential for pyrimidine biosynthesis and closely tied to respiratory chain function.

In amino acid metabolism, D-2-HG inhibits BCAT1/2, reducing glutamate production and increasing dependence on glutaminase. GLS inhibition sensitizes mIDH1 gliomas to oxidative stress and radiation therapy [161]. mIDH1 also impairs lipid biosynthesis through defective reductive carboxylation and NADPH depletion, leading to increased reliance on fatty acid β-oxidation. Targeting ACC1, via genetic knockdown, small-molecule inhibition, or dietary lipid restriction, disrupts lipid homeostasis and primes cells for apoptosis, thereby enhancing sensitivity to Venetoclax, a BCL-2 inhibitor, in AML and solid tumor models [59].

Alterations in glucose metabolism further compound these vulnerabilities. mIDH1 suppresses PDH activity, reducing mitochondrial glucose oxidation. Pharmacological activation of PDH with dichloroacetate (DCA) restores metabolic flux and inhibits proliferation in mIDH1 glioma models [162].

mIDH1 also disrupts DNA repair pathways, particularly homologous recombination (HR). D-2-HG increases repressive H3K9me3 at the ATM promoter, and KDM4 inhibition leads to global H3K9me3 accumulation, impairing HR factor recruitment [85, 163]. Inhibiting ALKBH2/3 and other HR enzymes further sensitizes mIDH1 cells to PARP inhibitors, which induce tumor regression in IDH-mutant gliomas and cholangiocarcinomas [164–166]. PARP inhibition is currently being tested in clinical trials for IDH-mutant tumors with HDAC inhibitors enhancing the effect by downregulating BRCA1 and RAD51 [167, 168].

Finally, mIDH1 increases vulnerability to ferroptosis and apoptosis. Ferroptosis sensitivity stems from impaired antioxidant defenses, including reduced glutathione synthesis and elevated lipid peroxidation. In HT-1080 fibrosarcoma models, inhibition of GPX4, a glutathione-dependent enzyme that blocks ferroptosis, led to selective killing of mIDH1 cells [169]. Additionally, mIDH1 AML exhibits increased dependence on the anti-apoptotic protein BCL-2, and the BCL-2 inhibitor venetoclax induces apoptosis and tumor regression in both in vitro and in vivo models [170].

Together, these synthetic lethal strategies expand the therapeutic landscape of mIDH1 cancers, offering rational combinations with IDH inhibitors or alternative therapies when resistance emerges, even as some vulnerabilities persist or evolve after 2-HG suppression.

### α-KG—Clinical and preclinical implications

While 2-HG acts as an oncometabolite, its precursor α-KG supports tumor suppression through roles in DNA demethylation, redox balance, and oxygen sensing. Therapeutic strategies that elevate α-KG or mimic its effects have shown promise in limiting tumor progression and modulating immunity.

Inhibiting α-KG dehydrogenase (α-KGDH) with small molecules such as AA6 leads to α-KG accumulation, activation of TET enzymes, downregulation of EMT drivers, and reduced metastasis [171]. Knockdown of transketolase also raises α-KG levels, enhancing PHD2 activity and destabilizing HIF-1α, thereby impeding tumor adaptation to hypoxia [76, 172].

Dimethyl-α-KG (DMKG) has been shown to sensitize triple-negative breast cancer (TNBC) to immune checkpoint blockade post-radiotherapy. In mouse models, the combination of DMKG, radiotherapy, and anti–PD-L1 therapy enhanced tumor suppression and increased apoptosis, supporting the use of α-KG analogs to reprogram the immune microenvironment [173].

CPI-613 (Devimistat), a lipoic acid analog that inhibits α-KGDH and PDH, disrupts mitochondrial metabolism and activates the AMPK-ATF3 axis. In melanoma, this boosted PD-L1 expression and enhanced the response to anti-PD-1 therapy [174].

CPI-613 has shown encouraging results in early-phase trials for AML and pancreatic cancer [175, 176], although a Phase III trial in metastatic pancreatic cancer (AVENGER 500) did not meet its primary endpoint [176]. In biliary tract cancer, CPI-613 combined with gemcitabine and cisplatin showed a 45% response rate and 10-month median PFS in the BilT-04 trial [177].

### Glutamine and α-KG – clinical implications

Glutamine is a critical nutrient for tumor cells, feeding the TCA cycle through its conversion to α-KG. Inhibiting enzymes along this axis, glutaminase (GLS) and glutamate dehydrogenase (GDH), has emerged as a strategy to suppress tumor growth and alter immune responses.

BPTES, an early GLS allosteric inhibitor, showed antitumor activity in preclinical models but faced challenges due to poor pharmacokinetics [178]. To address these issues, researchers have developed radiolabelled BPTES analogs, such as [^11^C]BPTES, to study their biodistribution and facilitate future clinical translation [179]. Telaglenastat (CB-839), a potent oral GLS1 inhibitor, has been tested in multiple clinical trials. While it improved PFS in combination with everolimus in RCC (ENTRATA trial) [180], the Phase II CANTATA trial failed to meet its endpoint [181]. Telaglenastat has shown tolerability and modest activity in TNBC, NSCLC, and hematologic malignancies [182–184], with potential benefit in tumors harboring KEAP1 or NRF2 mutations.

Targeting GDH offers an alternative approach. Inhibitors such as epigallocatechin-3-gallate (EGCG) and R162 suppress tumor growth in cholangiocarcinoma, glioma, and hepatocellular carcinoma [185–188]. EGCG has been explored in trials for colorectal (NCT02891538), ovarian (NCT00721890), and liver (NCT06015022) cancers, where it may help prevent recurrence or delay progression, although issues like poor bioavailability remain a challenge [189].

### Itaconate – therapeutic implications

Itaconate, a TCA cycle–derived immunometabolite produced by aconitate decarboxylase 1 (ACOD1/IRG1), has diverse roles in inflammation, microbial defense, and tumor biology. Initially characterized for its anti-inflammatory effects in macrophages, itaconate has more recently emerged as a modulator of tumor immunity. In murine models, genetic loss of ACOD1 improved responses to immune checkpoint blockade, suggesting that endogenous itaconate may suppress antitumor immunity under certain conditions [22, 190]. This positions the itaconate pathway as a metabolic checkpoint with therapeutic relevance in cancer.

Pharmacologically, citraconate, a structural analog of itaconate, binds ACOD1 to reduce endogenous itaconate levels and also acts as a strong electrophile that activates the NRF2–SLC7A11 antioxidant pathway more effectively than other isomers [191]. In obesity-associated models, elevated miR-144 reduces IRG1 expression, thereby increasing SDH and FH activity in hepatocytes. While this has not been directly observed in tumors, the finding implies that metabolic reprogramming by miRNAs could indirectly influence the itaconate axis [192]. While not yet validated in cancer, these findings highlight the potential for microRNA-driven metabolic rewiring of this pathway. Moreover, the transcription factor TFEB enhances mitochondrial itaconate synthesis in macrophages during bacterial infection, aiding host defense. Whether this TFEB–itaconate axis is active in tumors remains unknown but represents a compelling direction for future investigation [193].

Therapeutically, dimethyl itaconate (DI), a cell-permeable derivative, has shown anti-inflammatory and antitumor activity in colitis-associated colorectal cancer models. DI treatment reduced IL-1β and CCL2 expression in epithelial cells, limited infiltration of macrophages and myeloid-derived suppressor cells (MDSCs), and enhanced CD8⁺ T cell–mediated immunity. It also alleviated colitis symptoms, suggesting potential dual benefits in tumor control and inflammation management [194].

While the effects of itaconate are context-dependent, the pathway offers a promising therapeutic target in immune- and inflammation-driven cancers. Defining when to inhibit versus activate itaconate signalling will be critical, but emerging evidence supports its potential to complement immunotherapy and metabolic interventions.

## Conclusion and final remarks

Oncometabolites Such as 2-hydroxyglutarate, succinate, and fumarate are emerging as key regulators of cancer progression and immune evasion. By disrupting epigenetic landscapes, redox balance, and immune cell function, these metabolites reshape both tumor-intrinsic programs and the tumor microenvironment. Their influence extends across cell types, enabling immune suppression and therapy resistance.

Targeting oncometabolite-producing pathways has shown clinical promise, especially in IDH-mutant cancers. Ongoing efforts to combine metabolic inhibitors with immunotherapies may further enhance treatment responses. However, the heterogeneity of tumor metabolism, potential off-target effects, and resistance mechanisms remain major challenges.

Future research should focus on defining the context-specific roles of oncometabolites, their uptake and function in non-tumor cells, and how they interact with immune and stromal components. Emerging tools such as single-cell analysis, spatial transcriptomics, and metabolic imaging will be crucial in uncovering new therapeutic vulnerabilities. A deeper understanding of these metabolites could enable the design of precision strategies that target both the tumor and its immune contexture, ultimately improving cancer outcomes.

## Data Availability

No datasets were generated or analysed during the current study.

## Abbreviations

2-HG: 2-Hydroxyglutarate
α-KG: Alpha-ketoglutarate
α-KGDDs: Alpha-ketoglutarate-dependent dioxygenases
ACC1: Acetyl-CoA carboxylase 1
ACLY: ATP-citrate lyase
ACOD1: Aconitate decarboxylase 1
AEG-1: Astrocyte elevated gene-1
AKT: Protein kinase B
AML: Acute myeloid leukemia
AMPK: AMP-activated protein kinase
APC: Antigen-presenting cell
ATP: Adenosine triphosphate
B2M: Beta-2-microglobulin
BCAT1/2: Branched-chain amino acid transaminase ½
BCAA: Branched-chain amino acids
BCL-2: B-cell lymphoma 2
CIMP: CpG island methylator phenotype
CXCL10: C-X-C motif chemokine ligand 10
CTLA4: Cytotoxic T-lymphocyte-associated protein 4
DC: Dendritic cell
DHODH: Dihydroorotate dehydrogenase
DI: Dimethyl itaconate
DMKG: Dimethyl alpha-ketoglutarate
DMM: Dimethyl malonate
DNMT1: DNA methyltransferase 1
EGCG: Epigallocatechin-3-gallate
ETC: Electron transport chain
FAD: Flavin adenine dinucleotide
FH: Fumarate hydratase
FOXP3: Forkhead box P3
FTO: Fat mass and obesity-associated protein
G6PD: Glucose-6-phosphate dehydrogenase
GABA: Gamma-aminobutyric acid
G-CSF: Granulocyte colony-stimulating factor
GLS: Glutaminase
GLUD1: Glutamate dehydrogenase 1
GOT2: Glutamate oxaloacetate transaminase 2
GPR91: G-protein coupled receptor 91 (SUCNR1)
GSH: Glutathione
HCC: Hepatocellular carcinoma
HDAC: Histone deacetylase
HIF: Hypoxia-inducible factor
HR: Homologous recombination
ICC: Intrahepatic cholangiocarcinoma
IDH: Isocitrate dehydrogenase
IFN: Interferon
IL: Interleukin
iNKT: Invariant natural killer T (cell)
iTreg: Induced regulatory T cell
JAK: Janus kinase
KDM4: Lysine demethylase 4
LDH: Lactate dehydrogenase
m6A: N6-methyladenosine
MCT1: Monocarboxylate transporter 1
MDSC: Myeloid-derived suppressor cell
MHC: Major histocompatibility complex
mIDH1: Mutant isocitrate dehydrogenase 1
mTOR: Mechanistic target of rapamycin
NAD⁺ / NADH / NADPH: Nicotinamide adenine dinucleotide forms
NAMPT: Nicotinamide phosphoribosyltransferase
NFAT: Nuclear factor of activated T cells
NF-κB: Nuclear factor kappa-light-chain-enhancer of activated B cells
NK: Natural killer (cell)
NPC: Nasopharyngeal carcinoma
NRF2: Nuclear factor erythroid 2–related factor 2
OE-D-2-HG: Octyl ester D-2-hydroxyglutarate
OE-L-2-HG: Octyl ester L-2-hydroxyglutarate
OXPHOS: Oxidative phosphorylation
PARP: Poly (ADP-ribose) polymerase
PD-1: Programmed cell death protein 1
PD-L1: Programmed death-ligand 1
PDH: Pyruvate dehydrogenase
PHD: Prolyl hydroxylase domain
PI3K: Phosphoinositide 3-kinase
PKM2: Pyruvate kinase M2
PPP: Pentose phosphate pathway
PRDM1: PR domain zinc finger protein 1
PTM: Post-translational modification
RCC: Renal cell carcinoma
RIG-I: Retinoic acid-inducible gene I
RNA-seq: RNA sequencing
ROS: Reactive oxygen species
SDH: Succinate dehydrogenase
SETDB1: SET domain bifurcated histone lysine methyltransferase 1
SLC13A3: Solute carrier family 13 member 3
SLC1A1: Solute carrier family 1 member 1
SLC7A11: Solute carrier family 7 member 11
STAT3: Signal transducer and activator of transcription 3
SUCNR1: Succinate receptor 1
TAM: Tumor-associated macrophage
TCA: Tricarboxylic acid
TCR: T cell receptor
TET: Ten-eleven translocation
TFEB: Transcription factor EB
TGF-β: Transforming growth factor beta
Th17: T helper 17 cells
TIN: Tumor-infiltrating neutrophil
TME: Tumor microenvironment
TNBC: Triple-negative breast cancer
Treg: Regulatory T cell
TRIM28: Tripartite motif-containing 28
UQCRC1: Ubiquinol-cytochrome c reductase core protein 1
VDAC1: Voltage-dependent anion channel 1
WT: Wild type
ZAP70: Zeta-chain-associated protein kinase 70
